# Hypoxia as a potential cause of dyspareunia

**DOI:** 10.1371/journal.pone.0281268

**Published:** 2023-04-17

**Authors:** Karel Hurt, Frantisek Zahalka, Michal Zikan, Jana Rackova, Ivana Rakovicova, Jakub Rakovic, Martin Halad

**Affiliations:** 1 Obstetrics and Gynaecology Dpt., Teaching Hospital Bulovka, First Faculty of Medicine, Charles University Prague, Prague, Czechia; 2 Sports Motoric Laboratory, Faculty of Physical Education and Sport, Charles University Prague, Prague, Czechia; 3 OBGYN Department Amedeana Prague, Prague, Czechia; 4 OBGYN Department Next Prague, Prague, Czechia; Dipartimento di Scienze Mediche e Chirugiche (DIMEC), Orsola Hospital, ITALY

## Abstract

Dyspareunia is genital pain before, during or after penile-vaginal sexual intercourse. The prevalence of dyspareunia ranges from 8 to 22%. Sexual intercourse concomitant with a pelvic organic lesion is likely to cause pain in most cases. However, in these cases, the pain depends not only on sexual intercourse. In its basic definition, dyspareunia in women is considered an idiopathic affection without a typical organic constitution. It is only present with penile-vaginal penetration. Long-term hypoxia in perineal muscles can cause muscle and perimuscular changes, leading to chronic pain not sufficiently responding to standard therapy. During the entrance examination to our previous study on dyspareunia, we noted significantly lower pulse oximetry levels in the perineal area of affected women. We aimed to compare pulse oximetry oxygen saturation (SpO2) of dyspareunia-affected women to healthy, pain-free women. A retrospective study was performed. The study participants were women who had participated in our previously published study on dyspareunia. This retrospective study was approved by the Ethical Committee. The study included 62 women: 31 dyspareunia-affected women in the treatment group and 31 healthy women in the control group. Method: During their examinations, women in the dyspareunia and control groups were measured for SpO2. The procedure was performed in the vulvo-perineal rear region, involving the commissure and the bulbospongiosus muscle. Median and mean SpO2 were compared between the treatment and control groups. Testing for sample size accuracy was performed retroactively. Results: There were 31 participants in each group. The SpO2 data were skewed and did not follow a Gaussian distribution. The Mann-Whitney U test was run to determine differences in perineum oximetry between the treatment group and controls. The median SpO2 was 91 in the treatment group and 92 in the control group. This difference was statistically significant, p = 0.002. Sample size accuracy was assured by post hoc calculation. Conclusions: Idiopathic dyspareunia is inherent in cohabitation muscle pain that standard therapy could not explain nor treat. We detected clinically meaningfully decreased levels of SpO2 in affected patients. We compared pelvic oximetry between dyspareunia-affected women in the treatment and control groups. This comparison showed significant hypoxia in the perineal muscle area (p = 0.002). Our results may help us understand the source of this pain and guide treatment accordingly.

## Introduction

Dyspareunia is defined as genital pain that occurs just before, during or after sexual intercourse. This term is used for both sexes, but for obvious reasons it is more frequently discussed in relation to women [1]. It was first described in ancient Egypt in the Ramesseum Papyrus, the oldest surviving illustrated Papyrus roll [2]. Its frequency often depends on the period in a woman’s lifetime. The frequency of dyspareunia ranges from 8–22%, according to different authors [3]. However, it is thought to be more frequent in women about 40 years of age [4]. Sexual intercourse concomitant with a pelvic organic lesion or wound is a probable cause of pain in most cases.

Another reason could be non-physical aspects of dyspareunia or sexual trauma. Moreover, sexual dysfunctions can cause dyspareunia. Pelvic pain could be present at the site of the pelvic inflammation, vaginal mucous inflammation, discharge, endometriosis, mucous atrophy, pelvic surgery or after other affections [5, 6]. However, in these cases the pain depends not only on penile-vaginal intercourse but has an organic origin [7–9]. In most cases it disappears after regular treatment of its origin. It could be better described as vulvodynia. In its essential definition dyspareunia in women is considered an idiopathic condition without a typical organic constitution, i.e. it is only present with penile-vaginal penetration and not followed by any other condition [1, 10]. This dysfunction is very often associated with painful spasms of the pelvic muscles [11, 12].

For this reason, the term “vaginism” was probably included in this group. This dysfunction is defined as a painful spasm of the pelvic muscles that completely anatomically disables vaginal penetration [8]. The complex was named GPPPD (genito-pelvic pain or penetration disorder) [13].

The intensity (level of dyspareunia) was first defined by Marinoff et al. [14]. The authors proposed a four-point scale ranging from 0–3 (0 = no pain during intercourse, 1 = pain during intercourse that does not prevent intercourse, 2 = pain during intercourse that interrupts intercourse and 3 = pain that prevents intercourse). The scale describes pain limitations during sexual intercourse. Despite proper diagnostics, determining a cause and treatment for dyspareunia remains problematic.

Pain associated with muscle, surroundings and interpenetrating fascial tissue seems to be one of the most common diagnoses reported in general practice. Studies have focused on functional principles, including an altered expression of muscle metabolism and enhancing the role of peripheral factors in persistent chronic pain [15–17]. Some studies have reported an increased concentration of end products of anaerobic glycolysis and impairing oxidative metabolism by local hypoxia, presented as an insufficient capacity for muscle recovery because of inflammation and metabolic pattern changes [18]. Long-term hypoxia in perineal muscles could cause muscle and perimuscular changes, resulting in chronic pain not adequately responding to standard therapy. During the entrance examination to our previous study on dyspareunia, we noted significantly lower pulse oximetry levels in the perineal area of affected women [15, 19]. We focused on this event to explain the reason for the muscle and perimuscular changes. Specifically, we aimed to compare pulse oximetry oxygen saturation (SpO2) of dyspareunia-affected women (treatment group) to healthy, pain-free women (controls).

## Participants and method

A retrospective study was performed to compare the treatment group with the controls. The study participants were women who took part in our previous study on dyspareunia conducted between 2017 and 2019, following a former feasibility study (previously published) [20].

All study procedures were conducted in accordance with the ethical standards of the institutional and research committees and with the 1964 Helsinki Declaration and its later amendments or comparable ethical standards. The study protocol was approved by the Ethical Committee of the Teaching Hospital at Charles University, Prague, and all patients gave their informed consent and confirmed their participation by signing a consent form. This retrospective study protocol was also approved by the Ethical Committee of the Teaching Hospital at Charles University, Prague.

### Participants

The treatment group included 31 dyspareunia-affected women aged 24–51 (mean age 40) years. This group’s mean body mass index (BMI) was 24.6. The control group comprised 31 women aged 25–52 (mean age 39) years. The mean BMI of the control group was 24.8. The controls, recruited from volunteers taking part in regular prevention examinations, had a medical history free of any known pathology. Both groups were similar in baseline demographic characteristics.

All participants in the treatment group were dyspareunia positive with no other disease. They had no pelvic organic reason for their condition, including hormonal changes for dyspareunia, physical changes or a history of sexual trauma. By the time this study was conducted, all the women had fulfilled the study criteria. The inclusion and exclusion criteria for dyspareunia group have been and are published elsewhere and are mentioned consequently for reader’s better comprehension.

#### Inclusion criteria for dyspareunia group

Inclusion criteria were based on completing all the following criteria of dyspareunia: painful penile-vaginal penetration without pelvic organic reasons primarily connected with pain, a score of >0 on the Marinoff Dyspareunia Scale (Marinoff), a score >0 on a visual analogue scale (VAS), age 20–75 years and a duration of dyspareunia >3 months during the past 6 months. To evaluate discomfort the primary measurement was the Marinoff scale for dyspareunia. In addition, the VAS served as a secondary measure of pain.

Patient benefits were unobtainable through other therapeutic approaches.

#### Exclusion criteria for dyspareunia group

The exclusion criteria were acute pelvic inflammation during the past 6 months, oncological disease within the past 5 years, a clinically significant haematological disease (e.g., haemophilia or other bleeding disorders), myocardial infarction or cardiac arrhythmia within the past 6 months, any serious metabolic disorder (e.g., diabetes with organic changes) and affection in an intended application area.

## Method

**Fig 1:**
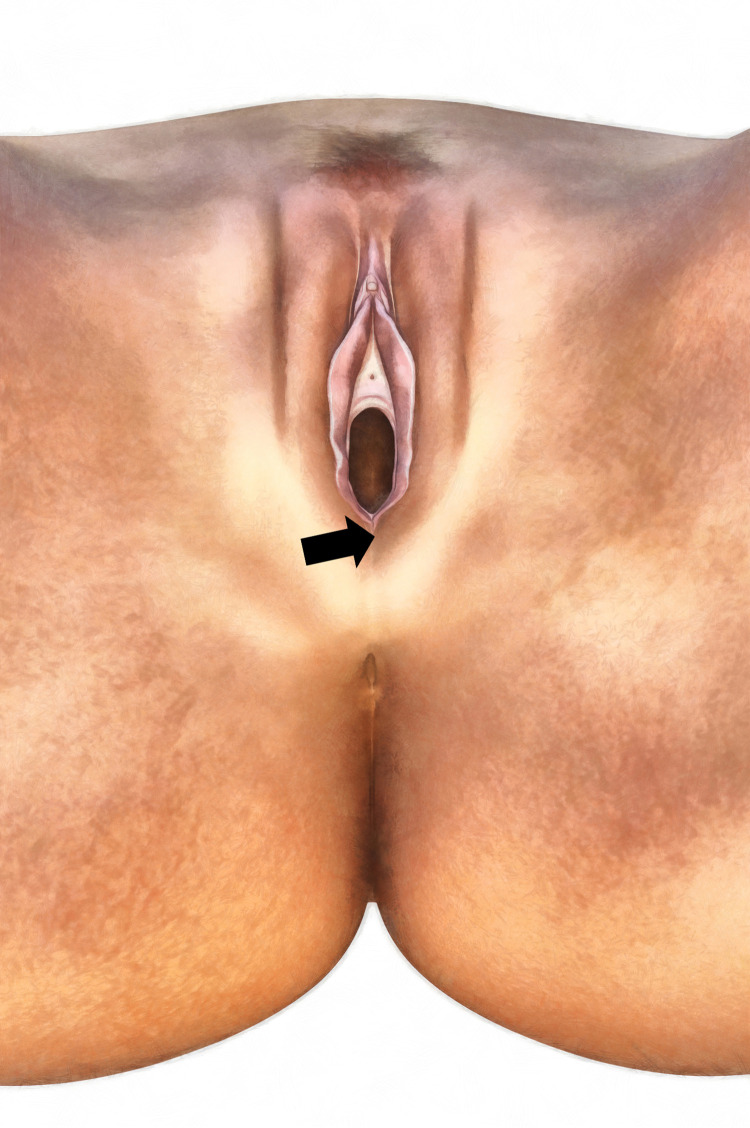
SpO_2_ measurement area in perineum region.

During their regular examinations, women in the treatment and control groups were measured for SpO2. The oximetry measurements were performed in the posterior commissure of the vagina between the mucous part of the vagina and the dermal part of the perineum, involving the bulbospongiosus muscle (Fig 1). The measurements were done in supine position with the biggest ear-clip sensor pulse oximeter and a plain vital functions measuring gadget. Measurement of SpO2 was recorded as a percentage. SpO2 is determined by measuring the absorption of two emitted light wavelengths, red at 660 nm and near-IR at 940 nm. The light transmitted through the tissue is detected by a photodiode on the opposite side. The pulse oximeter uses the relative amount of red and IR light absorbed to define the proportion of oxyhaemoglobin in deoxyhaemoglobin [19, 21, 22]. The ability of the pulse oximetry to detect SpO2 of only arterial blood is based on the principle that the amount of red and IR light absorbed fluctuates with the cardiac cycle as the arterial blood volume increases during systole and decreases during diastole. The blood volume in the capillaries, veins, fat, skin and bone remains relatively constant [19].

### Statistical analysis

Because of the nonparametric distribution of SpO2, The data were expressed as medians and ranges. All analyses were conducted using the IBM SPSS software package. We chose the nonparametric Mann-Whitney U test to compare the treatment (dyspareunia) and control (dyspareunia free)groups.

Sample size accuracy was determined retroactively using the IBM/Sample power analysis software.

## Results

There were 62 participants with 31 in each group (Table 1). The Shapiro-Wilk test confirmed that a Gaussian distribution of the variables was not met. The Mann-Whitney nonparametric U test was run to determine differences in perineum oximetry between the treatment and control groups. The distribution of SpO2 differed statistically between the two groups (p = 0.002). The median SpO2 was 91 in the treatment group and 92 in the control group. We computed the sample size to ensure that a two-sided test with α = 0.05 has 80% power to detect a true effect. With our data, 50 participants (25 per group) were needed, lower than the actual number of study participants (n = 62).

**Table 1:** Distribution of oximetry data in treatment and control groups.

Main characteristics of study participants: Treatment and Controls				
Perineal oximetry comparison of Treatment and Placebo groups				
by Mann-Whitney U test				
			Perineal	Oximetry
Group	Count	Age	Median	Range
Treatment	31	39.7	91	89_94
Controls	31	39.3	92	90_95
Mann-Whitney Asymptotic Significance (2-tailed) p = 0.002				
Treatment group = patients with dyspareunia				
Control group = healthy patients free of dyspareunia				
Median and Range = oximetry values expressed in %				

## Discussion

The present study confirms the presence of depressed oxygenation in the examined areas of the female perineum in dyspareunia-affected women. Due to local ischemia, abnormal tissue oxygenation is most likely present in the focused areas. The hypothesis was that a microcirculation change occurs in regions producing muscle pain [15]. Some authors have studied blood flow and reported a notable change in local microcirculation. They assumed that regulation of the microcirculation is disturbed in fibromyalgia, which could lead to sensitisation of the intramuscular nociceptors [18]. The lower density of capillaries in the focus area was also discussed. Histological tissue findings detected so called moth-eaten and ragged red fibres that may indicate mitochondria’s abnormal proliferation. This pathological distribution of mitochondria may be caused by local hypoxia from low blood flow in tender areas. A feasible alternative cause of hypoxia could be physical inactivity, which produces mitochondrial insufficiency and abnormal levels of critical metabolites for contractile muscle force [23]. Some authors refer to this phenomenon as deconditioning. Reduced daily activity causes deconditioning, affecting the overall picture of notable anatomical and biochemical defects in muscle [15, 24].

Yet, it should be noted that this study type of pain is only connected with cohabitation. If the pain is present at different time points or permanently, we have to describe the condition differently, probably as vulvodynia. We believe this is the first study expressly focusing on oximetry in the context of dyspareunia.

The strength of our study is that the technique used is painless for the patient, straightforward, low-cost and time efficient. Moreover, the procedure could be easily repeated without problems. It demands only standard vital function measuring unit with an adult ear probe to gather the data. The major disadvantage is the lack of standardisation for conducting this procedure. Another weakness of this study could be that it could overlook some diseases, which may bias the findings.

## Conclusions

Idiopathic dyspareunia is inherent in cohabitation muscle pain that standard therapy could neither explain nor treat. During the examination, we detected statistically significant decreased levels of SpO2 in our patient cohort. We compared pelvic oximetry between dyspareunia-affected and treated women and healthy controls (no dyspareunia). This comparison showed significant hypoxia in the perineal muscle area (p = 0.002). Our results may help understand the source of this pain and more accurately aim the treatment directly in the defective area.

### Protocol approval

Teaching hospital of Charles University Prague 3.8.2021/10150/EK-Z.
